# Smoking, body mass index, disease activity, and the risk of rapid radiographic progression in patients with early rheumatoid arthritis

**DOI:** 10.1186/s13075-018-1575-2

**Published:** 2018-05-02

**Authors:** Emil Rydell, Kristina Forslind, Jan-Åke Nilsson, Lennart T. H. Jacobsson, Carl Turesson

**Affiliations:** 1 Rheumatology, Department of Clinical Sciences, Malmö, Lund University, Jan Waldenströms gata 35, SE-202 13 Malmö, Sweden; 2 Department of Rheumatology, Skåne University Hospital, Inga Marie Nilssons gata 32, SE-214 28 Malmö, Sweden; 3 Department of Research and Education, Helsingborg Hospital, Charlotte Yhlens gata 10, SE-251 87 Helsingborg, Sweden; 4 Rheumatology, Department of Clinical Sciences, Helsingborg, Lund University, Svartbrödragränden 3–5, SE-251 87 Helsingborg, Sweden; 5 Department of Rheumatology and Inflammation Research, Sahlgrenska Academy at Gothenburg University, Guldhedsgatan 10 A, SE-405 30 Göteborg, Sweden

**Keywords:** Rheumatoid arthritis, Radiographic progression, Joint damage, Smoking, Body mass index, Disease activity

## Abstract

**Background:**

Identification of risk factors for rapid joint destruction in early rheumatoid arthritis (RA) can be helpful for optimizing treatment, and improving our understanding of destructive arthritis and its mechanisms. The objective of this study was to investigate the relationship between early RA patient characteristics and subsequent rapid radiographic progression (RRP).

**Methods:**

An inception cohort of patients with early RA (symptom duration < 12 months), recruited during 1995–2005 from a defined area (Malmö, Sweden), was investigated. Radiographs of the hands and feet were scored in chronological order according to the modified Sharp–van der Heijde score (SHS), by a trained reader. RRP was defined as an increase of ≥ 5 points in SHS per year.

**Results:**

Two hundred and thirty-three patients were included. Radiographs were available from 216 patients at baseline, 206 patients at 1 year, and 171 patients at 5 years. Thirty-six patients (22%) had RRP up to 5 years. In logistic regression models, rheumatoid factor (RF) and anti-cyclic citrullinated peptides (anti-CCP), and increased erythrocyte sedimentation rate (ESR) or C-reactive protein (CRP) at baseline, predicted RRP over 5 years. Patients identified as overweight or obese had a significantly reduced risk of RRP up to 5 years (odds ratio (OR) 0.26; 95% confidence interval (CI) 0.11–0.63; adjusted for RF, baseline erosions, and ESR). Similar point estimates were obtained when stratifying for antibody status, and in models adjusted for smoking. A history of ever smoking was associated with a significantly increased risk of RRP up to 5 years, independent of body mass index (BMI) (OR 3.17; 95% CI 1.22–8.28; adjusted for BMI). At the 1-year follow-up, erosive changes, Disease Activity Score of 28 joints, Health Assessment Questionnaire, swollen joint count, and patient’s global assessment of disease activity and pain were also significantly associated with RRP up to 5 years.

**Conclusions:**

A history of smoking, presence of RF and/or anti-CCP and early erosions, high initial disease activity and active disease at 1 year, all increase the risk of RRP. Patients with a high BMI may have a reduced risk of severe joint damage. This pattern was not explained by differences in disease activity or antibody status. The results of this study suggest independent effects of smoking and BMI on the risk of RRP.

## Background

In order to optimize treatment in patients with early rheumatoid arthritis (RA), with the intention to stop progression of joint destruction, it is important at an early stage to identify patients at high risk of rapid radiographic progression (RRP). Finding early risk factors is therefore of key importance. Previous research shows that patients with rheumatoid factor (RF) and anti-citrullinated protein antibodies (ACPA) have more severe disease with more extensive radiographic progression [1, 2]. Early joint erosions as well as high levels of markers of inflammation have also been associated with worse radiographic outcomes [3–6]. Environmental factors, such as smoking, may also affect the course of RA. Smoking is a risk factor for developing RA [7, 8], as well as for extra-articular manifestations in patients with established disease [9]. In addition, it has been suggested that smoking increases the risk for radiographic progression [10]. However, results from previous studies on smoking and radiographic damage are mixed [10–14]. Intricate relationships between smoking, antibodies, body composition, treatment response, and disease activity as well as differences in the design of previous studies make it difficult to assess the independent effect of smoking on the risk of radiographic progression.

Furthermore, although a high level of inflammation in the early stages of disease is a known risk factor for radiographic progression, the utility of the commonly used composite measure Disease Activity Score of 28 joints (DAS28) for identifying patients at risk and the importance of persistent disease activity are less clear [12, 15–17]. Previous research has indicated better outcomes for patients receiving disease-modifying antirheumatic drugs (DMARDs) at an early stage [18, 19]. However, such effects may vary across populations and depend on current treatment strategies.

Patients with high body mass index (BMI) have been shown in large studies to have higher disease activity [20, 21], to be less likely to respond to treatment [22], and to have more extensive disability [23]. Despite this, high BMI has been associated with reduced radiographic progression in a limited number of studies [24–28].

The purpose of this study was to investigate how patient characteristics, smoking status, disease activity measures over time, BMI, and time to initiation of DMARD treatment relate to subsequent RRP in patients with early RA.

## Methods

### Patients

An inception cohort of 233 consecutive patients with early RA was investigated. The catchment area was the city of Malmö, Sweden (population 259,579 in 2000). Patients were recruited from the rheumatology outpatient clinic of Malmö University Hospital, the only hospital serving the city, or from the four rheumatologists in private practice in the area, between 1995 and 2005.

The patients were diagnosed with RA by a specialist in rheumatology, fulfilled the 1987 American College of Rheumatology classification criteria for RA [29], and had a duration of symptoms ≤ 12 months at the time of inclusion. There were no additional exclusion criteria. All patients gave their written consent for participation in the study, including data collection and inclusion in the database. The study was approved by the Regional Ethical Review Board for southern Sweden (Lund, Sweden), and complied with the Declaration of Helsinki.

Results on clinical parameters and grip strength in a subset of the included patients have been reported previously [30].

### Clinical assessment

Patients were followed according to a structured program with evaluations at inclusion, 12 months, and 60 months. The same rheumatologist performed all of the clinical examinations. Patient characteristics and disease activity parameters were recorded, and radiographs of the hands and feet were obtained. The presence of erosions (present vs absent) was determined by a radiologist as part of standard clinical practice. Disability was assessed using the Swedish version of the Stanford Health Assessment Questionnaire (HAQ) [31]. Visual analogue scales (VASs) were used to evaluate the patients’ global assessment of disease activity and the patient’s assessment of pain. All patients were managed according to usual care with no prespecified protocol for antirheumatic treatment. The patients were included before the current practice of treat to target [32] was implemented, and before early treatment with biologic DMARDs came into widespread use. Information on height, weight, and smoking history (current/previous/never) was collected at inclusion through a self-administered questionnaire. For confirmation, information on smoking was also gathered through case-record reviews, with previous smoking being defined as a history of daily smoking for more than 6 months at anytime earlier in the patient’s life. The time from symptom onset to first start of DMARD treatment was assessed based on a review of medical records.

Data on treatment with biologic DMARDs at any time during the study period were obtained through linkage to a regional biologics register [33].

Changes in disease activity (DAS28) from baseline to the 1-year follow-up were categorized according to the European League Against Rheumatism (EULAR) response criteria [34].

### Laboratory investigations

RF and antibodies to cyclic citrullinated peptides (anti-CCP) were analyzed using standard ELISA methods at the immunology laboratories of the University Hospitals in Malmö and Lund. IgM RF was analyzed using ELISA, which was calibrated against the World Health Organization (WHO) RF reference preparation. Anti-CCP antibodies were analyzed using the Quanta Lite CCP IgG ELISA (INOVA Diagnostics, USA). The erythrocyte sedimentation rate (ESR) and C-reactive protein (CRP) were assessed according to standard methods at the Department of Clinical Chemistry, Malmö University Hospital.

### Radiographic assessment

Radiographs of the hands and feet were scored in chronological order according to the modified Sharp–van der Heijde score (SHS), by a trained reader (KF, coauthor) who was blinded to the clinical data. The intraclass correlation coefficient (ICC) from two readings with 2-week intervals for a subset of the cohort (*n* = 30) was 0.97. Based on the excellent ICC, a single reading was performed. The primary outcome, RRP, was defined as an increase of ≥ 5 points in SHS per year [35]. We also assessed SHS progression above the median as an outcome.

### Statistical analysis

Potential associations between each individual baseline variable with RRP over the first 5 years, as well as with above median progression of SHS during the same time period, were assessed using logistic regression analyses. Furthermore, the relation between 1-year variables and RRP between the follow-ups at 1 and 5 years was evaluated.

The covariates for the multivariate models were chosen based on the literature and the unadjusted analyses. Due to colinearity, we did not include both RF and anti-CCP. RF was chosen over anti-CCP due to the smaller number of patients with missing data for RF. The final multivariate analyses were adjusted for RF and for the presence of erosions according to standard radiologist assessment (independent of SHS scoring).

During part of the study period, high-sensitivity CRP analysis was not available and CRP values between 0 and 9 mg/l were reported by the laboratory as < 9 mg/l. In logistic regression models, CRP was therefore included as a dichotomized variable; that is, above versus below the median (9 mg/l) at inclusion and above versus below the 75th percentile (10 mg/l) at 1 year (since the median at 1 year was < 9 mg/l). Current smoking, previous smoking, and ever smoking were each compared to the reference category, never smoking.

BMI was included as a continuous variable. Furthermore, the risk of RRP in individuals fulfilling the WHO criteria for overweight or obesity (≥ 25 kg/m^2^), overweight (25–29.99 kg/m^2^), or obesity (≥ 30 kg/m^2^) was compared to that in individuals with normal BMI (18.5–24.99 kg/m^2^).

Predictors of SHS progression above the median were assessed in the same manner.

Statistical analysis was performed using IBM SPSS Statistics version 22.0 (IBM Corp. Armonk, NY, USA).

## Results

### Patient characteristics

A total of 233 patients with early RA (median symptom duration 7 months; interquartile range (IQR) 5–10) were included in this study. Characteristics at baseline and at the 1-year follow-up in patients with available radiographic data are presented in Table 1. A majority of the patients was treated with methotrexate (MTX) (Table 1), and 17% of all patients in the cohort (*n* = 40) were treated with a biologic DMARD at some time during the first 5 years. Among those with radiographic data, the most frequently used type of non-MTX DMARD was antimalarials (29% at inclusion, 20% at 1 year). Combination treatment (≥ 2 DMARDs) was used in three patients at baseline (2%) and in 13 patients (8%) at 1 year.

**Table 1:** Characteristics of patients with radiographic data

	Data available at inclusion and 5 years (*n* = 162)	Data available at 1 year and 5 years (*n* = 161)
	Characteristics at inclusion	Characteristics at 1 year
Demographics and history		
Female sex, *n* (%)	114 (70)	116 (72)
Age at inclusion (years)	62 (52–70)	62 (52–70)
Symptom duration at inclusion (months)	7 (5–10)	7 (5–10)
Time to first DMARD (months)^a^	5 (3–7)	5 (3–7)
Current treatment		
DMARD (any), *n* (%)	138 (85)	135 (84)
MTX, *n* (%)	85 (52)	97 (60)
MTX dose (mg/week)	10.0 (7.5–10.0)	10.0 (7.5–15.0)
Other DMARDs, *n* (%)	56 (35)	51 (32)
Concurrent prednisolone, *n* (%)	60 (37)	45 (28)
Prednisolone dose (mg/day)	7.5 (5.0–15.0)	5.0 (3.75–7.5)
Anthropometrics		
BMI (kg/m^2^)	25 (23–28)	NR
Obese^b^, *n* (%)	19 (12)	NR
Overweight^b^, *n* (%)	69 (45)	NR
Normal BMI^b^, *n* (%)	66 (43)	NR
Cigarette smoking status		
Current smokers, *n* (%)	49 (32)	NR
Previous smokers, *n* (%)	51 (33)	NR
Never smokers, *n* (%)	55 (36)	NR
Disease parameters		
RF-positive at inclusion, *n* (%)	105 (65)	104 (65)
Anti-CCP antibody-positive at inclusion, *n* (%)	83 (59)	80 (58)
Modified Sharp–van der Heijde score	2 (0–8)	6 (1–16)
Joint space narrowing score	0 (0–6)	4 (0–11)
Erosion score	0 (0–2)	2 (0–4)
Erosions present^c^, *n* (%)	28 (17)	47 (30)
DAS28	4.7 (3.6–5.7)	3.6 (2.7–4.4)
Remission^d^, *n* (%)	12 (8)	36 (23)
Low disease activity^d^, *n* (%)	27 (17)	56 (36)
Moderate disease activity^d^, *n* (%)	75 (47)	80 (51)
High disease activity^d^, *n* (%)	59 (37)	21 (13)
HAQ	0.75 (0.38–1.25)	0.50 (0.13–0.88)
Swollen joint count (out of 28)	7 (5–11)	4 (2–6)
Tender joint count (out of 28)	4 (2–9)	2 (0–5)
ESR (mm/h)	22 (11–43)	16 (8–30)
CRP (mg/l)^e^	9 (< 9–28)	< 9 (< 9–11)
Patient’s global assessment (VAS 0–100)	46 (21–65)	24 (11–48)
Pain (VAS 0–100)	40 (19–61)	24 (11–44)

Median (interquartile range) presented unless otherwise stated

For characteristics in patients with radiographic data available and included in the analysis for inclusion/1 year, missing numbers were as follows: symptom duration = 1/NA, time to DMARD = 15/NA, BMI = 5/NR, cigarette smoking status = 7/NR, RF = 1/1, anti-CCP = 22/22, erosion present = 1/2, DAS28 = 1/4, HAQ = 1/0, swollen joint count = 1/1, tender joint count = 1/1, ESR = 1/3, CRP = 1/2, patient’s global assessment = 1/2, pain = 1/2

*DMARD* disease-modifying antirheumatic drug, *MTX* methotrexate, *BMI* body mass index, *NR* not reported, *RF* rheumatoid factor, *anti-CCP* antibodies to cyclic citrullinated peptides, *DAS28* Disease Activity Score of 28 joints, *HAQ* Health Assessment Questionnaire, *ESR* erythrocyte sedimentation rate, *CRP* C-reactive protein, *VAS* visual analogue scale, *NA* not applicable

^a^Duration from rheumatoid arthritis symptom onset to start of first DMARD

^b^Definitions based on BMI: obese ≥ 30 kg/m^2^; overweight 25–29.99 kg/m^2^; normal 18.5–24.99 kg/m^2^. Three patients with BMI ≤ 18.5 kg/m^2^ were excluded from this analysis

^c^By standard radiographic evaluation, independent of modified Sharp–van der Heijde scoring

^d^Definitions based on DAS28: remission ≤ 2.6; low ≤ 3.2; moderate > 3.2 to ≤ 5.1; high > 5.1

^e^Analysis sensitivity differs, with some data ranging from 0 to 9 (mg/l) only reported as < 9 (mg/l)

### Radiographic progression

Radiographs were available for 216 patients at baseline, 206 patients at 1 year, and 171 patients at 5 years. Mean progression of SHS from baseline to 1 year, from baseline to 5 years, and from 1 year to 5 years was 4.0 (*n* = 194, standard deviation (SD) = 6.3), 17.4 (*n* = 162, SD = 20.0), and 13.8 (*n* = 161, SD = 19.0), respectively. Compared to baseline SHS values, 60 patients (31%) had RRP at 1 year (21 men, 39 women) and 36 patients (22%) up to 5 years (11 men, 25 women). Thirty-six patients (22%) had RRP from the 1-year follow-up to the 5-year follow-up (9 men, 27 women). The median SHS progression from baseline to 5 years was 12.

### Baseline predictors of RRP

Results of analyses of associations between baseline variables and RRP up to 5 years as well as above median progression in SHS up to 5 years are presented in Tables 2 and 3, respectively. Crude estimates and estimates adjusted for RF and baseline presence of erosions are presented. Age and sex were not significant predictors of RRP at 5 years in crude or adjusted analysis (Table 2).

**Table 2:** Baseline predictors of rapid radiographic progression up to 5 years

	Crude		Adjusted^a^	
	OR	95% CI	OR	95% CI
Demographics and anthropometrics				
Male sex	1.06	(0.47–2.37)	0.81	(0.34–1.89)
Age (per SD)^b^	1.20	(0.81–1.77)	1.34	(0.86–2.10)
Time to first DMARD (per SD)^b^	0.57	(0.25–1.30)	0.57	(0.23–1.42)
BMI (per SD)^b^	0.76	(0.51–1.15)	0.67	(0.44–1.03)
Normal BMI^c^ (reference)	1.00		1.00	
Obese^c^	0.10	(0.01–0.83)	0.07	(0.01–0.58)
Obese or overweight^c^	0.32	(0.15–0.71)	0.27	(0.12–0.63)
Overweight^c^	0.39	(0.18–0.88)	0.36	(0.15–0.84)
Smoking habits				
Never smoker (reference)	1.00		1.00	
Current smoker	3.60	(1.27–10.22)	2.92	(1.00–8.56)
Ever smoker	3.18	(1.22–8.24)	2.69	(1.01–7.18)
Previous smoker	2.79	(0.97–8.03)	2.50	(0.83–7.55)
Baseline disease parameters				
RF positivity	5.70	(1.90–17.10)	NA	NA
Anti-CCP positivity	6.04	(1.98–18.47)	3.69	(1.12–12.17)
Erosions present^d^	2.29	(0.95–5.53)	NA	NA
DAS28 (per SD)^b^	1.47	(0.99–2.18)	1.41	(0.94–2.11)
Disease activity^c^				
Low/moderate (reference)	1.00		1.00	
High	2.76	(1.29–5.89)	2.70	(1.21–6.03)
HAQ (per SD)^b^	1.37	(0.96–1.96)	1.55	(1.05–2.28)
ESR (per SD)^b^	1.89	(1.33–2.69)	1.70	(1.17–2.46)
CRP below median (reference)	1.00		1.00	
CRP above median (> 9 mg/l)	2.89	(1.31–6.39)	2.36	(1.04–5.38)
Swollen joint count (per SD)^b^	1.26	(0.87–1.84)	1.26	(0.86–1.86)
Tender joint count (per SD)^b^	0.85	(0.55–1.32)	0.94	(0.60–1.48)
Patient’s global assessment (VAS; per SD)^b^	1.36	(0.92–2.00)	1.29	(0.85–1.94)
Pain (VAS; per SD)^b^	1.16	(0.79–1.69)	1.18	(0.79–1.77)

*OR* odds ratio, *CI* confidence interval, *SD* standard deviation, *DMARD* disease-modifying antirheumatic drug, *BMI* body mass index, *NA* not applicable, *RF* rheumatoid factor, *anti-CCP* antibodies to cyclic citrullinated peptides, *DAS28* Disease Activity Score of 28 joints, *HAQ* Health Assessment Questionnaire, *ESR* erythrocyte sedimentation rate, *CRP* C-reactive protein, *VAS* visual analogue scale

^a^Adjusted for RF and presence of erosions

^b^SD: age 15 years; time to first DMARD 5.8 months; BMI 4.0 kg/m^2^; DAS28 1.4; HAQ 0.64; ESR 26 mm/h; swollen joint count 4.9; tender joint count 5.8; patient’s global assessment 26; pain 26

^c^For definitions see Table 1

^d^By standard radiographic evaluation, independent of modified Sharp–van der Heijde scoring

**Table 3:** Baseline predictors of radiographic progression with change in SHS above the median (i.e. > 12) up to 5 years

	Crude		Adjusted^a^	
	OR	95% CI	OR	95% CI
Demographics and anthropometrics				
Male sex	1.25	(0.64–2.46)	0.96	(0.46–1.98)
Age (per SD)^b^	1.18	(0.86–1.62)	1.23	(0.88–1.74)
Time to first DMARD (per SD)^b^	1.08	(0.80–1.45)	1.06	(0.74–1.53)
BMI (per SD)^b^	0.93	(0.67–1.29)	0.84	(0.59–1.19)
Normal BMI^c^ (reference)	1.00		1.00	
Obese^c^	0.98	(0.35–2.74)	0.79	(0.26–2.37)
Obese or overweight^c^	0.71	(0.37–1.34)	0.67	(0.33–1.34)
Overweight^c^	0.64	(0.33–1.27)	0.64	(0.31–1.33)
Smoking habits				
Never smoker (reference)	1.00		1.00	
Current smoker	2.16	(0.99–4.73)	1.82	(0.80–4.16)
Ever smoker	1.83	(0.93–3.57)	1.55	(0.76–3.16)
Previous smoker	1.56	(0.72–3.37)	1.39	(0.61–3.17)
Baseline disease parameters				
RF positivity	3.18	(1.60–6.34)	NA	NA
Anti-CCP positivity	2.47	(1.24–4.94)	1.41	(0.64–3.13)
Erosions present^d^	4.00	(1.59–10.06)	NA	NA
DAS28 (per SD)^b^	1.07	(0.78–1.47)	1.06	(0.75–1.49)
Disease activity^c^				
Low/moderate (reference)	1.00		1.00	
High	1.44	(0.76–2.75)	1.44	(0.72–2.86)
HAQ (per SD)^b^	0.99	(0.73–1.34)	1.08	(0.78–1.49)
ESR (per SD)^b^	1.73	(1.23–2.44)	1.57	(1.10–2.24)
CRP below median (reference)	1.00		1.00	
CRP above median (> 9 mg/l)	2.07	(1.11–3.88)	1.64	(0.84–3.19)
Swollen joint count (per SD)^b^	0.94	(0.68–1.30)	0.93	(0.66–1.32)
Tender joint count (per SD)^b^	0.61	(0.42–0.89)	0.66	(0.45–0.98)
Patient’s global assessment (VAS; per SD)^b^	1.16	(0.84–1.60)	1.13	(0.80–1.60)
Pain (VAS; per SD)^b^	0.99	(0.72–1.36)	1.03	(0.73–1.44)

*OR* odds ratio, *CI* confidence interval, *SD* standard deviation, *DMARD* disease-modifying antirheumatic drug, *BMI* body mass index, *NA* not applicable, *RF* rheumatoid factor, *anti-CCP* antibodies to cyclic citrullinated peptides, *DAS28* Disease Activity Score of 28 joints, *HAQ* Health Assessment Questionnaire, *ESR* erythrocyte sedimentation rate, *CRP* C-reactive protein, *VAS* visual analogue scale

^a^Adjusted for RF and presence of erosions

^b^SD: age 15 years; time to first DMARD 5.8 months; BMI 4.0 kg/m^2^; DAS28 1.4; HAQ 0.64; ESR 26 mm/h; swollen joint count 4.9; tender joint count 5.8; patient’s global assessment 26; pain 26

^c^For definitions see Table 1

^d^By standard radiographic evaluation, independent of modified Sharp–van der Heijde scoring

### Disease severity

Positive RF was a significant predictor of RRP over 5 years (odds ratio (OR) 5.23; 95% confidence interval (CI) 1.73–15.86; adjusted for baseline erosions). Significant associations for baseline variables with RRP up to 5 years were seen in adjusted models for anti-CCP positivity, ESR, and CRP (Table 2). No significant associations were observed for DAS28, HAQ, swollen and tender joint counts, VAS for patients’ global assessment of disease activity or pain, or time from symptom onset to DMARD initiation, when analyzed as continuous variables, for RRP up to 5 years (Table 2). Separate analysis of the baseline category of disease activity revealed a significantly increased risk of RRP up to 5 years for patients with high baseline DAS28 (> 5.1) compared to those with low to moderate disease activity (Table 2).

Baseline presence of erosions tended to predict RRP up to 5 years in unadjusted analysis (OR 2.29; 95% CI 0.95–5.53) (Table 2).

Results of analyses of predictors of SHS progression above the median (Table 3) were largely similar to the main results.

### Smoking and BMI

Significant associations with RRP from baseline up to 5 years were observed for current and ever smoking in crude and adjusted models (Table 2). Similar point estimates for the impact of ever smoking on the risk of RRP were obtained in analyses additionally adjusted for ESR (OR 2.69; 95% CI 0.98–7.44) or for DAS28 (OR 2.51; 95% CI 0.93–6.76). The pattern was similar for current smoking.

Obese or overweight patients had a reduced risk of RRP up to 5 years compared to those with normal BMI, with a numerically stronger effect for those who fulfilled the criteria for obesity (Table 2). The estimated impact of overweight/obesity (BMI > 25 kg/m^2^) on RRP up to 5 years was similar in RF-positive (OR 0.27; 95% CI 0.11–0.65) and RF-negative (OR 0.27; 95% CI 0.03–2.80) patients, and also in analyses stratified for anti-CCP status (positive OR 0.25 (95% CI 0.09–0.68); negative OR 0.26 (95% CI 0.03–2.70)). The presence of overweight/obesity was associated with a significantly reduced risk of RRP up to 5 years in analyses adjusted for RF and presence of erosions at baseline (Table 2), and also when additionally adjusting for ESR (OR 0.26; 95% CI 0.11–0.63). There were no major differences in baseline CRP, ESR, or DAS28 across categories of BMI (data not shown). In analyses stratified by sex, the negative association for overweight/obesity reached statistical significance in men (OR 0.17; 95% CI 0.04–0.74), with a similar trend in women (OR 0.43; 95% CI 0.17–1.07).

In multivariate analyses, adjusted for BMI, current smoking (OR 3.54; 95% CI 1.24–10.13) and ever smoking (OR 3.17; 95% CI 1.22–8.28) were both predictive of RRP over 5 years. Overweight/obesity was negatively associated with 5-year RRP, adjusted for ever smoking (OR 0.29; 95% CI 0.13–0.67), with a similar trend in analysis adjusted for current smoking (OR 0.44; 95% CI 0.16–1.23).

There were no significant associations between smoking or overweight/obesity and SHS progression above the median (Table 3).

### One-year variables as predictors of RRP

Disease activity parameters at 1 year had a significant impact on subsequent RRP up to 5 years (Table 4). Not only high ESR and CRP, but also DAS28, HAQ, swollen joint count, VAS global, and VAS pain analyzed as continuous variables, as well as the presence of erosions at the 1-year radiographic evaluation, were significantly associated with RRP up to 5 years in crude and adjusted models (Table 4). Additional analyses of erosive changes during the first year revealed that progression of SHS, total change in SHS, as well as RRP up to 1 year also significantly predicted RRP up to 5 years, in unadjusted models (Table 4). Numerically, presence of erosions, RRP during first year, CRP > 10 mg/l, and high/moderate disease activity at 1 year were the strongest predictors. Correspondingly, decreasing disease activity at 1 year, demonstrated by a greater change in DAS28 from baseline to 1 year or defined according to the EULAR response criteria as a good response at 1 year, drastically decreased the risk of RRP between the 1-year and 5-year follow-ups (Table 5).

**Table 4:** One-year predictors of rapid radiographic progression up to 5 years

	Crude		Adjusted^a^	
	OR	95% CI	OR	95% CI
1-year disease parameters				
Erosions present^b^	6.16	(2.77–13.73)	NA	NA
First-year progression (≥ 1 unit increase in SHS)	5.25	(1.90–14.47)	NA	NA
First-year change in SHS (per SD)^c^	2.31	(1.51–3.54)	NA	NA
RRP during first year	6.87	(2.98–15.82)	NA	NA
DAS28 (per SD)^c^	2.89	(1.81–4.61)	2.54	(1.54–4.19)
Disease activity^d^				
Low (reference)	1.00		1.00	
Moderate	7.13	(2.02–25.13)	6.14	(1.68–22.40)
High	13.25	(3.11–56.44)	9.05	(1.91–42.84)
HAQ (per SD)^c^	1.62	(1.12–2.33)	1.75	(1.18–2.61)
ESR (per SD)^c^	2.82	(1.77–4.50)	2.10	(1.30–3.37)
CRP below 75th percentile (reference)	1.00		1.00	
CRP above 75th percentile (> 10 mg/l)	10.32	(4.44–24.00)	6.98	(2.85–17.14)
Swollen joint count (per SD)^c^	1.97	(1.35–2.87)	1.79	(1.18–2.70)
Tender joint count (per SD)^c^	1.49	(1.01–2.19)	1.46	(0.94–2.29)
Patient’s global assessment (VAS; per SD)^C^	1.59	(1.08–2.34)	1.71	(1.10–2.66)
Pain (VAS; per SD)^c^	1.71	(1.16–2.52)	1.86	(1.19–2.91)

*OR* odds ratio, *CI* confidence interval, *NA* not applicable, *SHS* Sharp–van der Heijde score, *SD* standard deviation, *RRP* rapid radiographic progression, *DAS28* Disease Activity Score of 28 joints, *HAQ* Health Assessment Questionnaire, *ESR* erythrocyte sedimentation rate, *CRP* C-reactive protein, *VAS* visual analogue scale

^a^Adjusted for rheumatoid factor and presence of erosions

^b^By standard radiographic evaluation, independent of modified SHS

^c^SD: first year change in SHS 6.3; DAS28 1.3; HAQ 0.58; ESR 19 mm/h; swollen joint count 4.0, tender joint count 4.1; patient’s global assessment 23; pain 23

^d^For definitions see Table 1

**Table 5:** DAS28 response at 1 year and risk of rapid radiographic progression up to 5 years

	Crude		Adjusted^a^	
	OR	95% CI	OR	95% CI
Change in DAS28 (per SD)^b^	0.52	(0.34–0.81)	0.53	(0.33–0.86)
EULAR response^c^				
No response (reference)	1.00		1.00	
Moderate response	0.54	(0.24–1.21)	0.58	(0.24–1.41)
Good response	0.06	(0.01–0.49)	0.08	(0.01–0.64)
Moderate response (reference)	1.00		1.00	
Good response	0.12	(0.01–0.93)	0.14	(0.02–1.14)

*DAS28* Disease Activity Score of 28 joints, *OR* odds ratio, *CI* confidence interval, *SD* standard deviation, *EULAR* European League Against Rheumatism

^a^Adjusted for rheumatoid factor and presence of erosions at 1 year

^b^Standard deviation: 1.6

^c^Moderate response *n* = 61 (39%); good response *n* = 33 (21%)

## Discussion

In this inception cohort study, over 20% of patients with early RA had a substantial radiographic progression over the first 5 years of follow-up. There was a reduced risk of RRP in overweight and obese patients, and smoking was predictive of RRP, independent of BMI. These exposures appeared to affect the risk of rapid progression, rather than minor joint damage, as they were not significantly associated with SHS progression above the median (12 units over 5 years) in this study. Our results on the predictive value of seropositivity, erosions and high inflammatory markers at baseline are in agreement with the literature [36].

Previous research on the influence of smoking on radiographic progression has been somewhat inconclusive. Several studies have indicated an association between smoking and worse radiographic outcomes [10–12, 37–40], while some have shown no such association [13, 14, 41, 42]. Differences in adjustments for other possible contributors to joint damage, such as RF, ACPA, and disease activity, when examining the effects of smoking on radiographic progression limit comparability between studies. Another concern is the effect of smoking on body composition and BMI, which could be of importance in this context. However, our study suggests that smoking and BMI have independent effects on radiographic outcomes in RA.

The finding that patients with high BMI at inclusion were less likely to have rapidly progressive joint damage is consistent with previous research in the field [24–28]. In the Swedish SWEFOT study, a similar negative association between obesity and SHS progression over 2 years was observed, although it did not reach significance in the fully adjusted model, which included current smoking [43]. Apart from the SWEFOT study, only one other previously published study on this subject included adjustment for smoking [25], whereas others did not [24, 26–28]. In previous studies, the association with high BMI and less radiographic progression was found only to be significant among seropositive patients [25, 26]. Although the statistical power was limited for subanalyses of the RF or ACPA-negative patients in the present study, crude estimates on the impact of overweight/obesity in these subsets were similar to seropositive patients.

A recent study demonstrated a negative association between BMI and MRI-detected synovitis and bone marrow edema in patients with RA, while the reverse was observed for other types of arthritis [44]. This suggests that high BMI is specifically associated with downregulation of destructive arthritis in RA. The underlying pathways could be related to differences in adipokine production [45, 46], or other metabolic or hormonal factors, and should be furthered studied. The worse clinical symptoms observed in obese RA patients [47] may be due to other mechanisms including nonspecific pain, comorbidities, and immobility.

The associations for high baseline disease activity and failure to reduce DAS28 within 1 year with RRP are in accordance with some [12, 15, 17, 48], but not all [16, 49], previous studies. Differences in follow-up and in baseline disease activity and rates of radiographic progression may explain these discrepancies. Taken together, the ability to identify patients at higher risk of radiographic progression may improve when analyzing DAS28 over time, as both patients with high initial disease activity and those with active disease 1 year after diagnosis appear to be more prone to developing severe radiographic damage. Finally, early development of joint damage was a strong predictor of RRP.

In our study, time to initiation of DMARD treatment was not associated with RRP. Previous studies have shown that early treatment may alter the long-term course of disease and is of great importance in order to reduce radiographic progression over time [50]. Generally, short disease duration and small individual differences in time to initiation of treatment in our cohort are possible explanations for our results.

Limitations in this study include the relatively small sample size, which affects statistical power for the multivariate analyses. As data on smoking and BMI were only available at baseline, longitudinal evaluation of the impact of these factors was not possible. Since high-sensitivity CRP was not available during part of the follow-up, we could only estimate the impact of CRP by treating it as a dichotomous variable (see Statistical analysis section for further details).

Strengths of our study include the structured longitudinal follow-up of an inception cohort from a defined catchment area. Therefore, selection bias is not a major issue in this study, and the results could be generalized to patients with RA seen in clinical practice.

## Conclusion

A history of smoking, presence of RF and/or anti-CCP and early erosions, high initial disease activity and active disease at 1 year, all increase the risk of RRP. Patients with a high BMI may have a reduced risk of severe joint damage. This pattern was not explained by differences in disease activity or antibody status. The results of this study suggest independent effects of smoking and BMI on the risk of RRP.

## Abbreviations

ACPA: Anti-citrullinated protein antibodies
Anti-CCP: Antibodies to cyclic citrullinated peptides
BMI: Body mass index
CI: Confidence interval
CRP: C-reactive protein
DAS28: Disease Activity Score of 28 joints
DMARD: Disease-modifying antirheumatic drug
ESR: Erythrocyte sedimentation rate
EULAR: European League Against Rheumatism
HAQ: Health Assessment Questionnaire
IQR: Interquartile range
MTX: Methotrexate
OR: Odds ratio
RA: Rheumatoid arthritis
RF: Rheumatoid factor
RRP: Rapid radiographic progression
SD: Standard deviation
SHS: Sharp–van der Heijde score
VAS: Visual analogue scale
WHO: World Health Organization

## References

[1] van Steenbergen HW, Ajeganova S, Forslind K, Svensson B, van der Helm-van Mil AH (2015). The effects of rheumatoid factor and anticitrullinated peptide antibodies on bone erosions in rheumatoid arthritis. Ann Rheum Dis.

[2] Forslind K, Ahlmen M, Eberhardt K, Hafstrom I, Svensson B, BARFOT Study Group (2004). Prediction of radiological outcome in early rheumatoid arthritis in clinical practice: role of antibodies to citrullinated peptides (anti-CCP). Ann Rheum Dis.

[3] Combe B, Dougados M, Goupille P, Cantagrel A, Eliaou JF, Sibilia J (2001). Prognostic factors for radiographic damage in early rheumatoid arthritis: a multiparameter prospective study. Arthritis Rheum.

[4] Visser K, Goekoop-Ruiterman YP, de Vries-Bouwstra JK, Ronday HK, Seys PE, Kerstens PJ (2010). A matrix risk model for the prediction of rapid radiographic progression in patients with rheumatoid arthritis receiving different dynamic treatment strategies: post hoc analyses from the BeSt study. Ann Rheum Dis.

[5] Tobon G, Saraux A, Lukas C, Gandjbakhch F, Gottenberg JE, Mariette X (2013). First-year radiographic progression as a predictor of further progression in early arthritis: results of a large national French cohort. Arthritis Care Res (Hoboken).

[6] Lindqvist E, Eberhardt K, Bendtzen K, Heinegard D, Saxne T (2005). Prognostic laboratory markers of joint damage in rheumatoid arthritis. Ann Rheum Dis.

[7] Silman AJ, Newman J, MacGregor AJ (1996). Cigarette smoking increases the risk of rheumatoid arthritis. Results from a nationwide study of disease-discordant twins. Arthritis Rheum.

[8] Bergstrom U, Jacobsson LT, Nilsson JA, Berglund G, Turesson C (2011). Pulmonary dysfunction, smoking, socioeconomic status and the risk of developing rheumatoid arthritis. Rheumatology (Oxford).

[9] Nyhall-Wåhlin BM, Petersson IF, Nilsson JA, Jacobsson LT, Turesson C, BARFOT study group (2009). High disease activity disability burden and smoking predict severe extra-articular manifestations in early rheumatoid arthritis. Rheumatology (Oxford).

[10] Ruiz-Esquide V, Gomez-Puerta JA, Canete JD, Graell E, Vazquez I, Ercilla MG (2011). Effects of smoking on disease activity and radiographic progression in early rheumatoid arthritis. J Rheumatol.

[11] de Rooy DP, van Nies JA, Kapetanovic MC, Kristjansdottir H, Andersson ML, Forslind K (2014). Smoking as a risk factor for the radiological severity of rheumatoid arthritis: a study on six cohorts. Ann Rheum Dis.

[12] Saevarsdottir S, Rezaei H, Geborek P, Petersson I, Ernestam S, Albertsson K (2015). Current smoking status is a strong predictor of radiographic progression in early rheumatoid arthritis: results from the SWEFOT trial. Ann Rheum Dis.

[13] Westhoff G, Rau R, Zink A (2008). Rheumatoid arthritis patients who smoke have a higher need for DMARDs and feel worse, but they do not have more joint damage than non-smokers of the same serological group. Rheumatology (Oxford).

[14] Vesperini V, Lukas C, Fautrel B, Le Loet X, Rincheval N, Combe B (2013). Association of tobacco exposure and reduction of radiographic progression in early rheumatoid arthritis: results from a French multicenter cohort. Arthritis Care Res (Hoboken).

[15] Svensson B, Andersson M, Forslind K, Ajeganova S, Hafstrom I, BARFOT study group (2016). Persistently active disease is common in patients with rheumatoid arthritis, particularly in women: a long-term inception cohort study. Scand J Rheumatol.

[16] Fautrel B, Granger B, Combe B, Saraux A, Guillemin F, Le Loet X (2012). Matrix to predict rapid radiographic progression of early rheumatoid arthritis patients from the community treated with methotrexate or leflunomide: results from the ESPOIR cohort. Arthritis Res Ther.

[17] Welsing PM, Landewe RB, van Riel PL, Boers M, van Gestel AM, van der Linden S (2004). The relationship between disease activity and radiologic progression in patients with rheumatoid arthritis: a longitudinal analysis. Arthritis Rheum.

[18] Kyburz D, Gabay C, Michel BA, Finckh A, physicians of SCQM-RA (2011). The long-term impact of early treatment of rheumatoid arthritis on radiographic progression: a population-based cohort study. Rheumatology (Oxford).

[19] Mottonen T, Hannonen P, Korpela M, Nissila M, Kautiainen H, Ilonen J (2002). Delay to institution of therapy and induction of remission using single-drug or combination-disease-modifying antirheumatic drug therapy in early rheumatoid arthritis. Arthritis Rheum.

[20] Jawaheer D, Olsen J, Lahiff M, Forsberg S, Lahteenmaki J, da Silveira IG (2010). Gender, body mass index and rheumatoid arthritis disease activity: results from the QUEST-RA Study. Clin Exp Rheumatol.

[21] Ajeganova S, Andersson ML, Hafström I, BARFOT Study Group (2013). Association of obesity with worse disease severity in rheumatoid arthritis as well as with comorbidities: a long-term followup from disease onset. Arthritis Care Res (Hoboken).

[22] Heimans L, van den Broek M, le Cessie S, Siegerink B, Riyazi N, Han KH (2013). Association of high body mass index with decreased treatment response to combination therapy in recent-onset rheumatoid arthritis patients. Arthritis Care Res (Hoboken).

[23] Giles JT, Bartlett SJ, Andersen RE, Fontaine KR, Bathon JM (2008). Association of body composition with disability in rheumatoid arthritis: impact of appendicular fat and lean tissue mass. Arthritis Rheum.

[24] Kaufmann J, Kielstein V (2003). Relation between body mass index and radiological progression in patients with rheumatoid arthritis. J Rheumatol.

[25] Westhoff G, Rau R, Zink A (2007). Radiographic joint damage in early rheumatoid arthritis is highly dependent on body mass index. Arthritis Rheum.

[26] van der Helm-van Mil AH, van der Kooij SM, Allaart CF, Toes RE, Huizinga TW (2008). A high body mass index has a protective effect on the amount of joint destruction in small joints in early rheumatoid arthritis. Ann Rheum Dis.

[27] de Rooy DP, van der Linden MP, Knevel R, Huizinga TW, van der Helm-van Mil AH (2011). Predicting arthritis outcomes—what can be learned from the Leiden Early Arthritis Clinic?. Rheumatology (Oxford).

[28] Baker JF, Ostergaard M, George M, Shults J, Emery P, Baker DG (2014). Greater body mass independently predicts less radiographic progression on X-ray and MRI over 1-2 years. Ann Rheum Dis.

[29] Arnett FC, Edworthy SM, Bloch DA, McShane DJ, Fries JF, Cooper NS (1988). The American Rheumatism Association 1987 revised criteria for the classification of rheumatoid arthritis. Arthritis Rheum.

[30] Rydholm M, Book C, Wikström I, Jacobsson L, Turesson C. Despite early improvement, patients with rheumatoid arthritis still have impaired grip force 5 years after diagnosis. Arthritis Care Res (Hoboken). 2017;70:491–498. 10.1002/acr.23318. 10.1002/acr.23318 28692794

[31] Ekdahl C, Eberhardt K, Andersson SI, Svensson B (1988). Assessing disability in patients with rheumatoid arthritis. Use of a Swedish version of the Stanford Health Assessment Questionnaire. Scand J Rheumatol.

[32] Smolen JS, Aletaha D, Bijlsma JW, Breedveld FC, Boumpas D, Burmester G (2010). Treating rheumatoid arthritis to target: recommendations of an international task force. Ann Rheum Dis.

[33] Geborek P, Nitelius E, Noltorp S, Petri H, Jacobsson L, Larsson L (2005). Population based studies of biological antirheumatic drug use in southern Sweden: comparison with pharmaceutical sales. Ann Rheum Dis.

[34] van Gestel AM, Prevoo ML, van ‘t Hof MA, van Rijswijk MH, van de Putte LB, van Riel PL (1996). Development and validation of the European League Against Rheumatism response criteria for rheumatoid arthritis. Comparison with the preliminary American College of Rheumatology and the World Health Organization/International League Against Rheumatism Criteria. Arthritis Rheum.

[35] Vastesaeger N, Xu S, Aletaha D, St Clair EW, Smolen JS (2009). A pilot risk model for the prediction of rapid radiographic progression in rheumatoid arthritis. Rheumatology (Oxford).

[36] Carpenter L, Nikiphorou E, Sharpe R, Norton S, Rennie K, Bunn F, et al. Have radiographic progression rates in early rheumatoid arthritis changed? A systematic review and meta-analysis of long-term cohorts. Rheumatology (Oxford). 2016;55:1053–1065. 10.1093/rheumatology/kew004. 10.1093/rheumatology/kew004 26961746

[37] Mattey DL, Hutchinson D, Dawes PT, Nixon NB, Clarke S, Fisher J (2002). Smoking and disease severity in rheumatoid arthritis: association with polymorphism at the glutathione S-transferase M1 locus. Arthritis Rheum.

[38] Haye Salinas MJ, Retamozo S, Alvarez AC, Maldonado Ficco H, Dal Pra F, Citera G (2015). Effects of cigarette smoking on early arthritis: a cross-sectional study-data from the Argentine Consortium for Early Arthritis (CONAART). Rheumatol Int.

[39] Papadopoulos NG, Alamanos Y, Voulgari PV, Epagelis EK, Tsifetaki N, Drosos AA (2005). Does cigarette smoking influence disease expression, activity and severity in early rheumatoid arthritis patients?. Clin Exp Rheumatol.

[40] Saag KG, Cerhan JR (1997). Cigarette smoking and rheumatoid arthritis severity. Ann Rheum Dis.

[41] Manfredsdottir VF, Vikingsdottir T, Jonsson T, Geirsson AJ, Kjartansson O, Heimisdottir M (2006). The effects of tobacco smoking and rheumatoid factor seropositivity on disease activity and joint damage in early rheumatoid arthritis. Rheumatology (Oxford).

[42] Quintana-Duque MA, Rondon-Herrera F, Calvo-Paramo E, Yunis JJ, Varela-Narino A, Iglesias-Gamarra A (2017). The impact of smoking on disease activity, disability, and radiographic damage in rheumatoid arthritis: is cigarette protective?. Rheumatol Int.

[43] Levitsky A, Brismar K, Hafstrom I, Hambardzumyan K, Lourdudoss C, van Vollenhoven RF (2017). Obesity is a strong predictor of worse clinical outcomes and treatment responses in early rheumatoid arthritis: results from the SWEFOT trial. RMD Open.

[44] Mangnus L, Nieuwenhuis WP, van Steenbergen HW, Huizinga TW, Reijnierse M, van der Helm-van Mil AH (2016). Body mass index and extent of MRI-detected inflammation: opposite effects in rheumatoid arthritis versus other arthritides and asymptomatic persons. Arthritis Res Ther.

[45] Giles JT, Allison M, Bingham CO, Scott WM, Bathon JM (2009). Adiponectin is a mediator of the inverse association of adiposity with radiographic damage in rheumatoid arthritis. Arthritis Rheum.

[46] Meyer M, Sellam J (2013). Serum level of adiponectin is a surrogate independent biomarker of radiographic disease progression in early rheumatoid arthritis: results from the ESPOIR cohort. Arthritis Res Ther.

[47] Vidal C, Barnetche T, Morel J, Combe B, Daien C (2015). Association of body mass index categories with disease activity and radiographic joint damage in rheumatoid arthritis: a systematic review and metaanalysis. J Rheumatol.

[48] Landewe R, van der Heijde D, Klareskog L, van Vollenhoven R, Fatenejad S (2006). Disconnect between inflammation and joint destruction after treatment with etanercept plus methotrexate: results from the trial of etanercept and methotrexate with radiographic and patient outcomes. Arthritis Rheum.

[49] Rezaei H, Saevarsdottir S, Forslind K, Albertsson K, Wallin H, Bratt J (2012). In early rheumatoid arthritis, patients with a good initial response to methotrexate have excellent 2-year clinical outcomes, but radiological progression is not fully prevented: data from the methotrexate responders population in the SWEFOT trial. Ann Rheum Dis.

[50] Finckh A, Liang MH, van Herckenrode CM, de Pablo P (2006). Long-term impact of early treatment on radiographic progression in rheumatoid arthritis: a meta-analysis. Arthritis Rheum.

